# Time Course of Mitochondrial Antioxidant Markers in a Preclinical Model of Severe Penetrating Traumatic Brain Injury

**DOI:** 10.3390/ijms26030906

**Published:** 2025-01-22

**Authors:** Sudeep Musyaju, Hiren R. Modi, Deborah A. Shear, Anke H. Scultetus, Jignesh D. Pandya

**Affiliations:** Brain Trauma Neuroprotection (BTN) Branch, Walter Reed Army Institute of Research (WRAIR), Silver Spring, MD 20910, USA

**Keywords:** penetrating traumatic brain injury, mitochondrial dysfunction, antioxidants, free radicals, oxidative stress, time course

## Abstract

Traumatic brain injury (TBI) results from external mechanical forces exerted on the brain, triggering secondary injuries due to cellular excitotoxicity. A key indicator of damage is mitochondrial dysfunction, which is associated with elevated free radicals and disrupted redox balance following TBI. However, the temporal changes in mitochondrial redox homeostasis after penetrating TBI (PTBI) have not been thoroughly examined. This study aimed to investigate redox alterations from 30 min to two-weeks post-injury in adult male Sprague Dawley rats that experienced either PTBI or a Sham craniectomy. Redox parameters were measured at several points: 30 min, 3 h, 6 h, 24 h, 3 d, 7 d, and 14 d post-injury. Mitochondrial samples from the injury core and perilesional areas exhibited significant elevations in protein modifications including 3-nitrotyrosine (3-NT) and protein carbonyl (PC) adducts (14–53%, vs. Sham). In parallel, antioxidants such as glutathione, NADPH, peroxiredoxin-3 (PRX-3), thioredoxin-2 (TRX-2), and superoxide dismutase 2 (SOD2) were significantly depleted (20–80%, vs. Sham). In contrast, catalase (CAT) expression showed a significant increase (45–75%, vs. Sham). These findings indicate a notable imbalance in redox parameters over the two-week post-PTBI period suggesting that the therapeutic window to employ antioxidant therapy extends well beyond 24 h post-TBI.

## 1. Introduction

Traumatic brain injury (TBI) is a leading cause of death and disability across all ages globally. Owing to its growing prevalence, the Centers for Disease Control and Prevention (CDC) has referred to TBI as a silent epidemic. About one-third of all injury-related deaths in the United States are caused by TBI [1]. Military service members are at the highest risk of this silent epidemic due to their engagement in combat and training exposures. Civilian injuries are often due to gunshots, with devastating consequences. Unfortunately, most causes of TBI are unpredictable and not preventable, and research efforts are underway to identify and evaluate treatment options for the management of TBI. In this line of effort, further research on understanding TBI pathogenesis, identifying novel molecular targets, and establishing effective treatment strategies are much needed to improve TBI outcomes. One of the barriers in understanding and potentially improving post-injury outcomes is the lack of comprehensive knowledge of post-injury time-dependent molecular events, which regulate the advancement of TBI pathogenesis after trauma.

In addition to heterogeneity in the patient population, the complex and progressive nature of TBI involves a multi-dimensional cascade of primary and secondary responses leading to altered cognitive and behavioral outcomes. The neurologic damage produced by the primary traumatic mechanical forces is not alterable. However, the neurologic impairment caused by a cascade of secondary events after mechanical trauma can be mitigated and to some extent be reversible by targeting molecular events during the early period. Much of our understanding of the multiphasic pathobiology of TBI have arisen from animal models that simulate features of human TBI. Several pre-clinical TBI models, including penetrating TBI (PTBI), controlled cortical impact (CCI), blast (BTBI), and closed head injury (CHI), have ascertained that mitochondrial dysfunction is a shared immediate common indicator of cellular damage [2,3,4,5] that may play a pivotal role in secondary excitotoxic post-injury events. Mitochondria-centric key cellular mechanisms involved are calcium homeostasis, energy homeostasis, and redox homeostasis. Their imbalance subsequently may prompt downstream cellular processes such as pro-apoptotic pathway activation and neuronal death and alter behavior outcomes in TBI. Multiple published reports highlight models and cellular mechanisms of TBI [6,7,8,9,10,11].

In the injured brain, exploring redox targets and the therapeutic potential of antioxidants to mitigate secondary injury remains an area of interest. Therefore, detection and quantification of several redox markers have been evaluated as an indicator for pathological processes of TBI. As a byproduct of respiration, a mitochondria-centric increase in free radicals, such as reactive oxygen species (ROS) and reactive nitrogen species (RNS), evolves over time. The ROS-RNS are classically defined as partially reduced metabolites of oxygen that possess strong oxidizing capabilities [12], and mitochondria remain as the key mediator of free radical-driven redox homeostasis in the healthy brain [13,14,15,16]. A delicate balance between free radicals and various endogenous antioxidants is crucial for maintaining normal physiological functions of the brain. TBI perturbs redox homeostasis and provokes complex neuropathological events in the brain [17,18]. While the mitochondrial respiratory chain is the primary component that produces intracellular ROS, the antioxidant system is activated as a primary line of defense to control and quench ROS-RNS accumulation and avoid cell destruction [19]. Intracellular ROS-RNS levels are balanced by an intricate array of antioxidants. This includes compounds like glutathione and nicotinamide adenine dinucleotide phosphate (NADPH), as well as enzymes such as catalase (CAT) and superoxide dismutase (SOD), which catalyze reactions aimed at the elimination of excess ROS-RNS. Additionally, other endogenous antioxidants, such as peroxiredoxins-3 (PRX-3) and thioredoxins-2 (TRX-2), ensure critical monitoring of cellular ROS-RNS levels and conserve normal redox homeostasis.

In TBI, each of these aforementioned neuroprotective antioxidants in the brain become overwhelmed, resulting in oxidative cell damage [20]. When the enzymatic antioxidant system fails to counterbalance the excessive ROS production, brain cells produce oxidized lipids, proteins, and damaged DNA, ultimately leading to cell death and neuronal tissue damage [21]. ROS-induced protein oxidation leads to elevated protein oxidation with respect to 3-nitrotyrosine (3-NT) and protein carbonyl (PC) adduct formation. Additionally, under oxidative stress conditions, dysfunctional mitochondria cannot meet the energy demand of neuronal cells for their physiological functions; hence, they become vulnerable to rapid cell death [22]. Thus, oxidative stress as a mediator of secondary sequelae after TBI might be an essential factor contributing to long-term neurobehavioral deficits post-TBI. In this regard, oxidative stress following mitochondrial dysfunction can be referred to as a common denominator in the multi-mechanistic pathological cascade of secondary brain injury.

Although accumulating studies, including our previous studies [18,23,24], have implied that the concept of oxidative stress-induced secondary brain injury is central to understanding the pathobiology of TBI, the precise temporal course of oxidative stress responses at the cellular and subcellular levels after a penetrating TBI (PTBI) is currently unknown. The activation pattern, the role of oxidative stress, and downstream mechanisms for such protection following PTBI still need to be elucidated. To fill these knowledge gaps, the present investigation evaluates the comprehensive time course of oxidative stress and antioxidant markers after PTBI. We explored the redox homeostasis markers during acute to sub-acute phases using a rodent PTBI model with evaluations from 30 min to 14 days post-injury. Collectively, the current results provide a robust platform for evaluating mitochondrial-targeted therapeutics in experimental TBI.

## 2. Results

This study assessed the time course evolution of mitochondria-centric oxidative stress and antioxidant markers following PTBI. After PTBI or Sham injury, these markers were measured in the injury core and perilesional mitochondria at seven different time points between 30 min and 14 days post-injury. PTBI animals were sacrificed at corresponding time points for each experimental group together with Sham-operated animals (controls) included in all seven time points. Our primary focus was to verify mitochondria-centric changes in redox and antioxidant parameters; therefore, these parameters were tested in the mitochondrial (Mito) fraction. Additionally, glutathione content was quantified in the mitochondria (Mito), homogenate (Homo), and cytosol (Cyto) fractions, whereas NADPH content was quantified in the Mito fraction. The selection of assays were guided by our previous research, along with considerations of sensitivity, accuracy, equipment availability, and volume of sample required. Overall, this study illustrated the time-dependent imbalance of redox homeostasis and antioxidants parameters up to two weeks post-injury.

### 2.1. Temporal Changes in Mitochondrial Protein Oxidation Markers Post-PTBI

**Figure 1:**
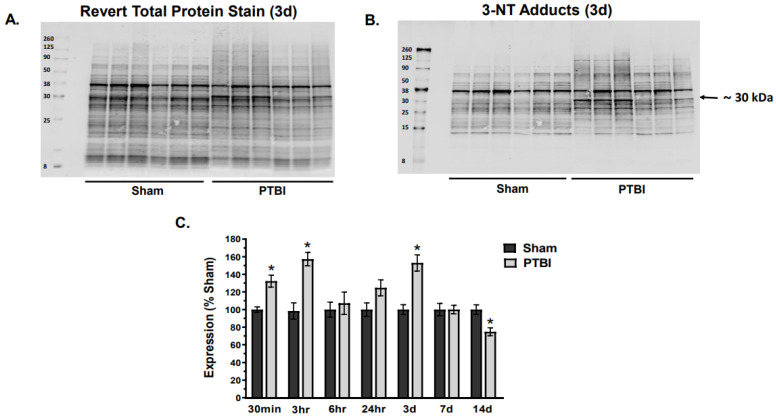
Temporal changes in mitochondrial 3-nitrotyrosine (3-NT) following PTBI. (**A**,**B**) Representative WB image of Revert total protein and 3-NT adduct formation at 3 d, respectively. 3-NT adducts were quantified between 8–260 kDa total protein bands and immuno-reactivity of 3-NT was observed at protein 15 kDa and higher. (**C**) Quantitative time course analysis of 3-NT histogram showed increased expression as early as 30 min after the injury and remained increased until 3 d. 3-NT expression level returned to Sham values at 7 d. There was a biphasic spike of 3-NT expression at 3 h and 3 d after PTBI compared to the Sham group values. Notably, a significant decrease in 3-NT was observed at 14 d. The values are presented in percentage change between the groups (Sham vs. PTBI). Bars represent each group’s mean ± SEM values (N = 6 animals per group). * *p* < 0.05 compared to the Sham control group (*t*-test).

**Figure 2:**
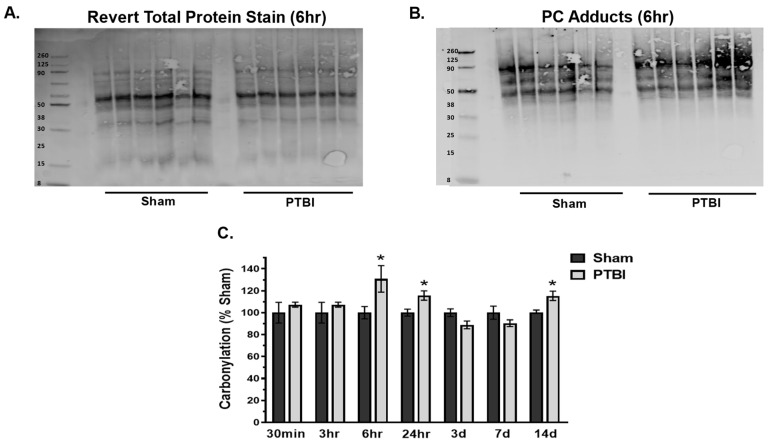
Temporal changes in mitochondrial protein carbonyls (PC) following PTBI. (**A**,**B**) Representative WB image of Revert total protein and PC adduct formation at 6 h, respectively. PC expression in mitochondria from the injury core was measured during the first 14 d post-PTBI period. (**C**) Quantitative histogram results of the time course of PC expression showed an increasing trend at 30 min of injury, with a significant increase seen at 6 h, and it remained significantly high until 24 h when compared with sham-operated animals. The difference between groups were non-significant between 3 d to 7 d followed by a significant spike of PC at 14 d post-injury period. Thus, PC exhibited biphasic spike at 24 h and 14 d after PTBI. The values are presented in percentage change between the groups (Sham vs. PTBI). Bars represent each group’s mean ± SEM values (N = 6 animals per group). * *p* < 0.05 compared to the Sham control group (*t*-test).

While most ROS-RNS are highly active moieties, it is challenging to measure their liberation directly due to their instability and short-lived nature; hence, we used the indirect method to detect the secondary effect of ROS-RNS liberation by detecting formation of protein oxidative adducts such as PC and 3-NT following PTBI. In this study, we employed the recently published RTP-based normalization method (Figure 1A and Figure 2A), which is a modified version of the traditional WB method for detecting protein oxidation and antioxidant quantification, in our present study [25].

In the first set of experiments, we sought to characterize the time course of oxidatively modified proteins following PTBI. The analysis revealed a time-dependent effect of PTBI on oxidative stress, as evidenced by elevated protein nitration and carbonylation. In the PTBI group, 3-NT expression demonstrated significant time-dependent changes with increasing values observed as early as 30 min (33%, * *p* < 0.05 vs. Sham) post-injury, and remained high until 3 d (53%, * *p* < 0.05 vs. Sham) (Figure 1B,C). This increase in 3-NT displayed a biphasic pattern, reaching over 57% of Sham-operated values at 3 h (* *p* < 0.05 vs. Sham), remained unchanged compared to Sham levels at 6 h, and was again elevated significantly at 3 d post-injury compared to Sham (Figure 1C). Similar to our earlier observation at 24 h, we observed that ∼30 kDa mitochondrial protein had remarkable protein nitration adduct formation following PTBI throughout the time course.

Interestingly, the protein carbonylation, an oxidation of the side chains of proteins to introduce ketone and aldehyde groups in a protein, also showed a biphasic response during a 2-week period, like 3-NT; however, this response had a late-onset. The PC level in the injured group revealed a significant first peak level at 6 h (32%, * *p* < 0.05 vs. Sham), and its elevation persisted at 24 h (Figure 2B,C). At 7 d post-injury, there was no significant difference between the injured and Sham groups. A second peak with a significant increase in PC content was observed at 14 d.

Collectively, protein oxidation can be seen as a contributing factor to secondary brain damage after TBI within the first two weeks.

### 2.2. Time Course of the First Line of Defense Antioxidant System Post-PTBI

**Figure 3:**
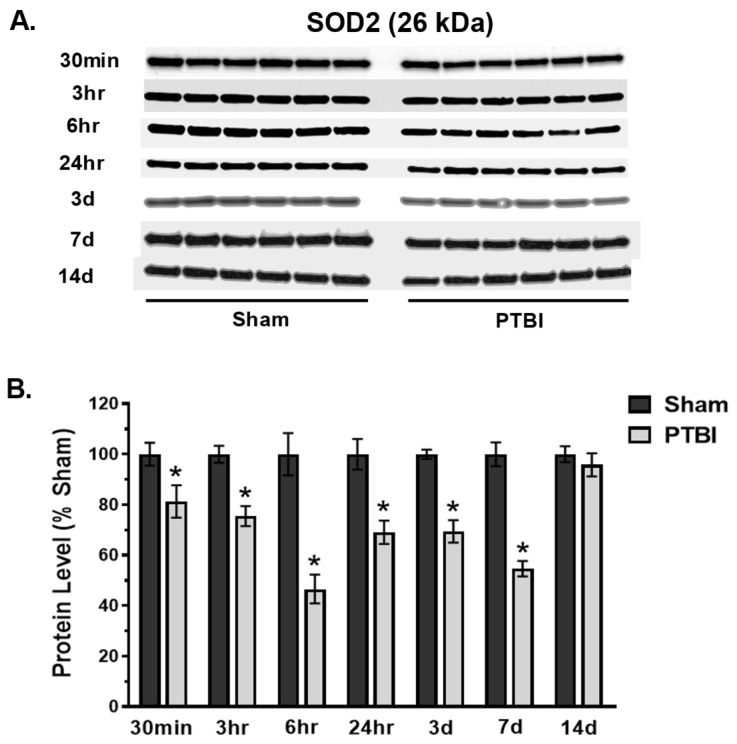
Time course of effects of TBI on mitochondrial SOD2. (**A**) Western blot images for SOD2 signals in mitochondria. SOD2 expressed at 26 kDa. (**B**) Quantitative histogram results of temporal profiling of SOD2 expression showed a significant decrease versus the Sham group after 30 min through 7 d. SOD2 expression returned to Sham levels by 14 d. The values are presented in percentage change between the groups (Sham vs. PTBI). Bars represent each group’s mean ± SEM values (N = 6 animals per group). * *p* < 0.05 compared to the Sham control group (*t*-test).

Next, we evaluated the temporal changes in the first line of antioxidant defense system that constitutes the manganese-dependent superoxide dismutase 2 (SOD2) and cataCAT following PTBI using the WB method. Analysis of SOD2 in the PTBI group demonstrated a significant time-dependent decrease in expression compared to Sham. The SOD2 expression at 26 kDa was significantly decreased starting at 30 min (20%, * *p* < 0.05 vs. Sham) post-injury, and it remained low until 7 d post-injury. Maximum depletion of SOD2 was observed between 6 h and 7 d (45–55%, * *p* < 0.05 vs. Sham) (Figure 3A,B). By 14 d post-injury, SOD2 levels in PTBI remained unchanged compared to Sham.

**Figure 4:**
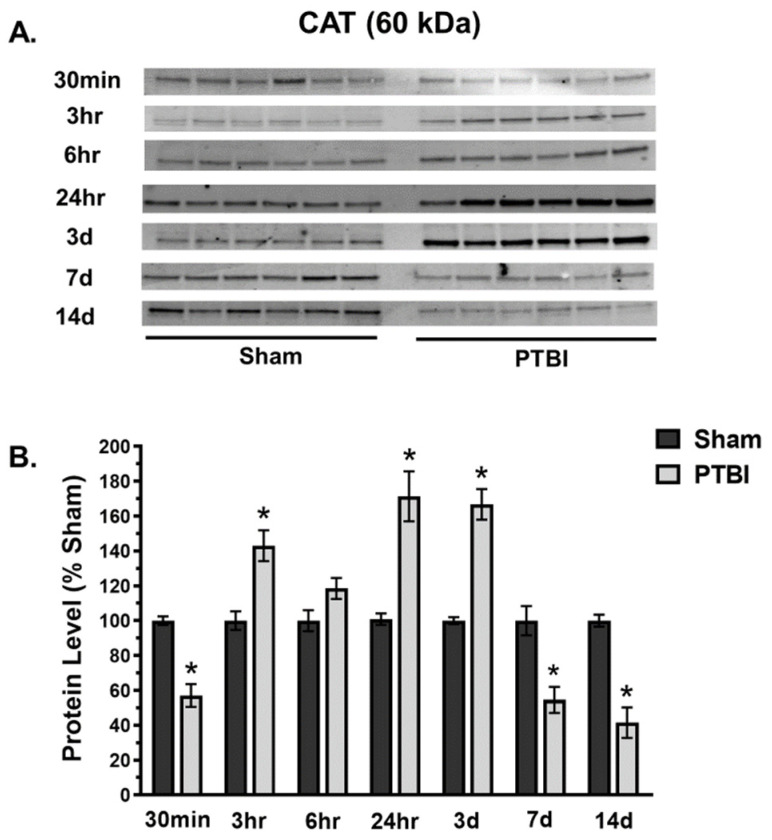
Time course of effects of TBI on mitochondrial CAT. (**A**) Western blot images for CAT signals in mitochondria. CAT expressed at 60 kDa. (**B**) Quantitative histogram results of temporal profiling of CAT exhibited a bell-shaped pattern. CAT was significantly increased following 3 h of injury and remained high until 3 d; a decrease in the CAT level was observed at 7 d, and it remained depleted until 14 d. The values are presented in percentage change between the groups (Sham vs. PTBI). Bars represent each group’s mean ± SEM values (N = 6 animals per group). * *p* < 0.05 compared to the Sham control group (*t*-test).

In contrast, CAT expression at 60 kDa initially decreased significantly at 30 min (47%, * *p* < 0.05 vs. Sham), then significantly increased at 3 h (45%, * *p* < 0.05 vs. Sham), and it remained high until 3 d of injury (70%, * *p* < 0.05 vs. Sham). Again, a decrease in the CAT level was observed at 7 d and it remained depleted at 14 d post-injury (Figure 4A,B).

Taken together, these data demonstrate a complex temporal spectrum of antioxidant homeostasis during two weeks of the post-injury period.

### 2.3. Temporal Changes in Thiol-Dependent Redoxins Post-PTBI

**Figure 5:**
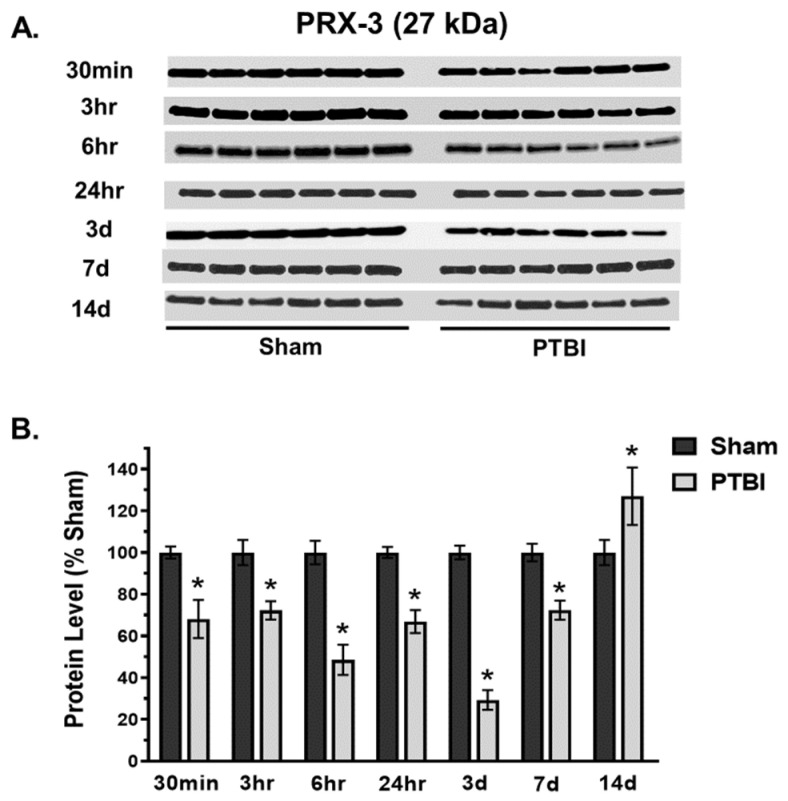
Time course of effects of TBI on mitochondrial PRX-3. (**A**) Western blot images for PRX-3 signals in mitochondria. PRX-3 expressed at 27 kDa. (**B**) Quantitative histogram results of time course of PRX-3 analysis demonstrated a significant time-dependent decrease in expression following injury. A significant decrease was observed as early as 30 min, and the depletion continued, with maximum deficit observed at 3 d. PRX-3 expression returned to Sham level by 14 d. The values are presented in percentage change between the groups (Sham vs. PTBI). Bars represent each group’s mean ± SEM values (N = 6 animals per group). * *p* < 0.05 compared to the Sham control group (*t*-test).

Additionally, the effect of TBI on an ubiquitous family of mitochondria-specific thiol-dependent antioxidant enzymes, such as PRX-3 and TRX-2 proteins, was evaluated. A significant decline in PRX-3 expressed at 27 kDa was observed as early as 30 min (33%, * *p* < 0.05 vs. Sham) after injury, and the depletion continued up to 7 d post-injury, with a maximum deficit observed at 3 d post-injury (71%, * *p* < 0.05 vs. Sham) (Figure 5A,B). The PRX-3 expression in the injured group was significantly upregulated at 14 d post-injury compared to Sham.

**Figure 6:**
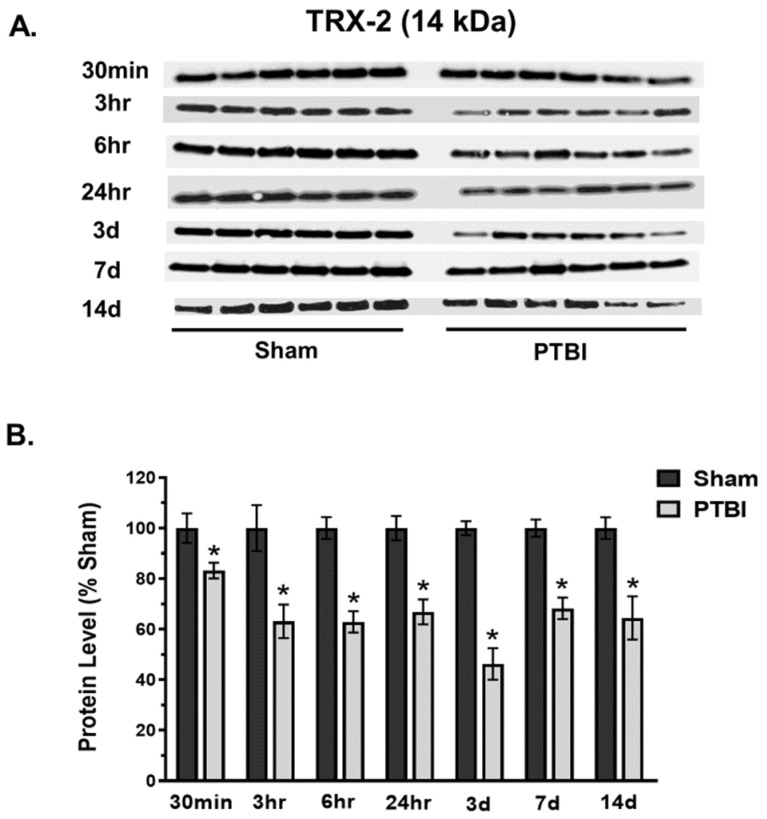
Time course of the effects of TBI on mitochondrial TRX-2. (**A**) Western blot images for TRX-2 signals in mitochondria. TRX-2 expressed at 14 kDa. (**B**) Quantitative histogram results of time course of PRX-3 analysis revealed a significant decline in TRX-2 as early as 30 min, and the depletion continued for 14 d. The maximum deficit was evident at 3 d. The values are presented in percentage change between the groups (Sham vs. PTBI). Bars represent each group’s mean ± SEM values (N = 6 animals per group). * *p* < 0.05 compared to the Sham control group (*t*-test).

Consistent with the results from other antioxidants, analysis of TRX-2 expression at 14 kDa demonstrated a significant time-dependent decrease in expression following PTBI. A significant decline in TRX-2 was observed as early as 30 min (18%, * *p* < 0.05 vs. Sham) after injury, and the depletion continued up to 14 d post-injury period, with a maximum deficit observed at 3 d post-injury (54%, * *p* < 0.05 vs. Sham) (Figure 6A,B).

### 2.4. Time Course of Principal Antioxidants Content Post-PTBI

**Figure 7:**
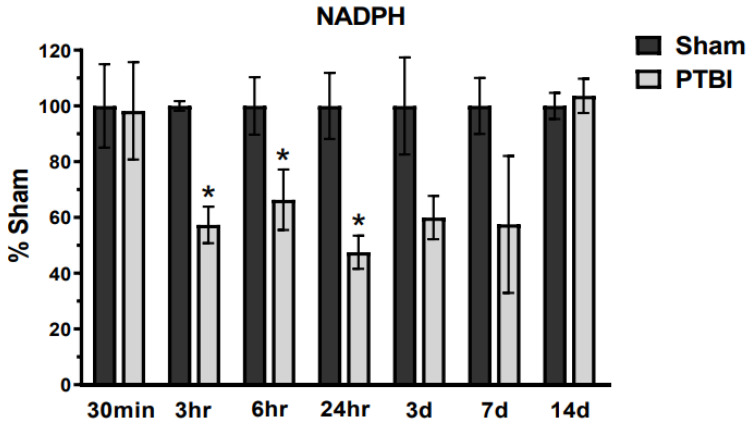
Time course of TBI-induced modifications in NADPH levels in the rodent brain: Quantitative histogram results of time course of NADPH showed a significant decrease starting at 3 h, and depletion of NADPH levels persisted for up to 7 d, with maximum depletion observed at 24 h. NADPH levels normalized to Sham levels at 14 d. The values are presented in percentage change between the groups (Sham vs. PTBI). Bars represent each group’s mean ± SEM values (N = 3–6 animals per group). * *p* < 0.05 compared to the Sham control group (*t*-test).

We assessed endogenous components of cellular metabolism, NADPH and Glutathione, equipped with crucial antioxidant properties. These antioxidants required unique extraction procedures; therefore, samples were collected from a separate cohort of animals (Table in Section 4.2) at respective time points. NADPH, which acts as a hydride (hydrogen anion) donor in a variety of antioxidant enzymatic processes, exhibited a significant decrease starting at 3 h (42%, * *p* < 0.05 vs. Sham) after PTBI, with depletion persisting for up to 7 d and normalizing to Sham levels at 14 d post-injury. The maximum decline was observed at 24 h post-PTBI (52%, * *p* < 0.05 vs. Sham) (Figure 7).

**Figure 8:**
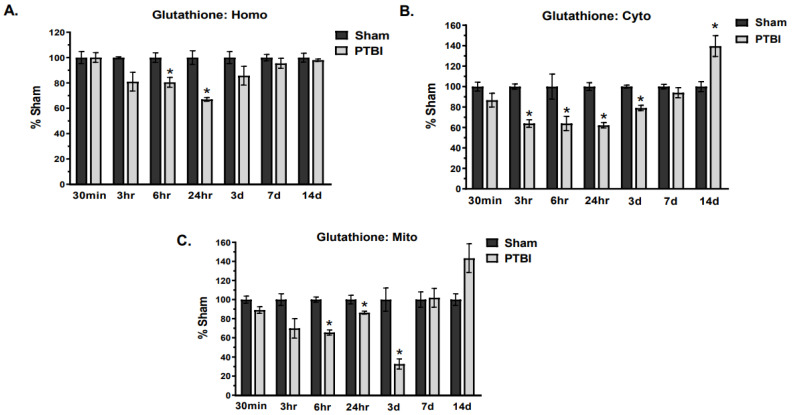
Time course of TBI-induced modifications of total glutathione content in the rodent brain: Glutathione content was measured in three cellular fractions (cytosol, homogenate, and mitochondria). (**A**,**B**) Quantitative histogram results of time course showed that glutathione content in cytosol and homogenate fractions were significantly decreased in the early insult periods (30 min to 3 d) after PTBI, with maximum depletion observed at 24 h, and their values returned to that of the Sham group at 7 d. Interestingly, cytosolic fraction showed a significant increase in glutathione level at 14 d. (**C**) Glutathione content in mitochondrial fraction exhibited a decreasing trend starting at 30 min, with significant depletion between 6 h and 3 d. Mitochondrial glutathione normalized to Sham values by 7 d, and it displayed a non-significant increasing trend at 14 d. The values are presented in percentage change between the groups (Sham vs. PTBI). Bars represent each group’s mean ± SEM values (N = 3–6 animals per group). * *p* < 0.05 compared to the Sham control group (*t*-test).

Similarly, the total glutathione content demonstrated a significant time-dependent downregulation. Considering that most of the cellular glutathione pool is present in the cytosol, the glutathione content was measured in three subcellular fractions (Homo, Cyto, and Mito) from PTBI and Sham groups. Homogenate showed a decrease in glutathione content starting at 3 h, with maximum depletion at 24 h (33%, * *p* < 0.05 vs. Sham) and returning to Sham values by 14 d. Similarly, cytosol showed a non-significant decrease in glutathione at 30 min post-injury, followed by a significant decline from 3 h up to 3 d, with a maximum depletion noted at 24 h post-injury (38%, * *p* < 0.05 vs. Sham). However, the value of glutathione in the cytosol fraction was significantly higher at 14 d compared to Sham. Consistent with this finding, mitochondrial fraction also showed a decreasing trend of glutathione as early as 30 min, with maximum depletion observed at 3 d (68%, * *p* < 0.05 vs. Sham). By 7 d, mitochondrial glutathione values returned to Sham levels, followed by a delayed spike observed at 14 d (Figure 8A–C).

Collectively, NADPH and glutathione showed a comparable pattern of impairment after PTBI, with depletion starting at 3 h and recovery occurring by 14 d.

## 3. Discussion

Oxidative stress is amongst the key mediators of the secondary injury cascade in TBI pathology. Our earlier time course study showed the robust biphasic nature of mitochondrial bioenergetics dysregulation detected up to 2 weeks post-injury following PTBI [26]. PTBI-induced mitochondrial dysfunction responses were time-specific and could evolve over weeks. Failure of mitochondrial homeostasis may contribute to the increased formation of free radicals, which are unstable atoms that trigger oxidative damage and exert noxious effects on the central nervous system (CNS). This conceptualization is also based on our most recent work, which demonstrated a significant redox imbalance at 24 h following PTBI, and is complementary with previously reported literature [18,23,27]. However, there remains a strong need for rigorous studies to understand the temporal profile of oxidative injury mechanisms following penetrating TBI, which may identify novel targets and treatment regimens duration to evaluate neuroprotective therapeutics. These considerations prompted us to perform a time course analysis of the oxidative stress and antioxidant markers to develop a comprehensive picture of PTBI’s impact on redox homeostasis at the mitochondrial level in the injured brain.

Notably, rodent models of TBI studies have shown mixed and conflicting redox effects [18,24,28,29,30,31,32,33,34]. TBI has been reported to decrease, increase, or induce no change in many oxidative stress markers compared to control in pre-clinical settings [18,24,28,29,30,31,32,33,34,35,36]. These discrepancies are likely related to TBI heterogeneity (contact vs. focal injury), injury severity (mild to severe), time points (acute to chronic period), and differential experimental methods/procedures used to quantify outcome. Although advances have been made to delineate the role of oxidative stress in TBI, yet many gaps remain unresolved. More comprehensive studies like the present one utilizing multiple post-injury time points are necessary to substantiate and clarify the dynamic nature of redox status progression following severe TBI. This study employing a military-relevant penetrating model of TBI has produced several important findings, as discussed systematically below.

**Figure 9:**
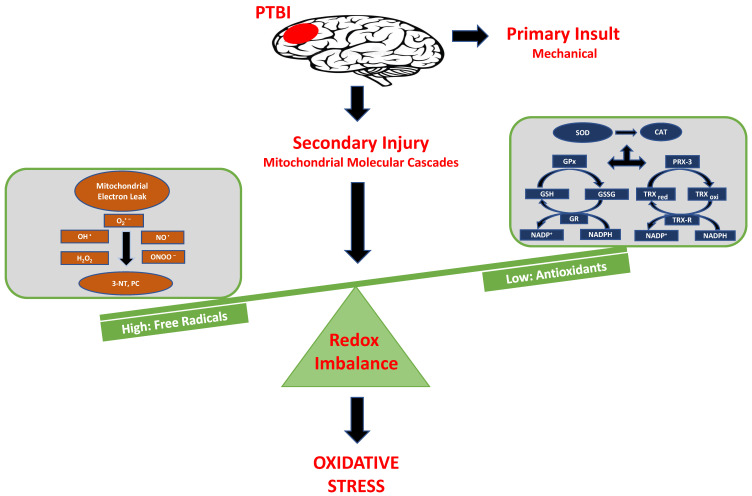
Key elements of mitochondria-centric redox imbalnce after TBI. Graphical representation of dynamics of key elements of mitochondria-centric redox imbalance, and oxidative stress following penetrating traumatic brain injury. PTBI is initiated by external mechanical assaults to the brain, which leads to secondary injury progression through cellular excitotoxic responses. PTBI further leads to mitochondria-centric processes including elevated free radicals driven protein oxidation (left-side of balance), and loss of endogenous antioxidants level (right-side of balance); further tip-off normal balance of redox homeostasis and leads to time-dependent elevated oxidative stress response.

Overall, our data herein demonstrated that the hallmark indicators of oxidative protein damage, 3-NT and PC expression, following PTBI were markedly augmented during the first few days of PTBI (Figure 1 and Figure 2). Second, the mitochondrial antioxidant defense that constitutes SOD2, PRX-3, and TRX-2 was significantly decreased in PTBI compared with Sham control over a 2-week period (Figure 3, Figure 4, Figure 5 and Figure 6). Third, levels of both NADPH, a universal redox cofactor, and glutathione, the most abundant non-protein thiol in cellular defense, were significantly depleted following PTBI compared with Sham for the immediate and up to 3 d post-injury time points (Figure 7 and Figure 8). Finally, our graphical abstract illustrates key elements of mitochondria-centric redox imbalance and oxidative stress following PTBI (Figure 9). Thus, our results suggest that redox hemostasis is severely compromised during the initial days of the TBI, probably as a part of the progressive secondary PTBI pathogenesis. This pathological phenomenon may originate from an overproduction of ROS and/or from a reduction in antioxidant defenses [15]. Our data aligns well with the large fraction of literature reporting elevated oxidative stress in severe models of TBI [14,18,24,37,38].

The imbalance between oxidants and antioxidants following TBI could indirectly affect the clearance of ROS, as indicated by the elevated levels of ROS byproducts 3-NT and PC in the present study. Our findings are in close agreement with previous studies showing elevated ROS in PTBI at 24 h post-PTBI [23], upregulation of mitochondrial 3-NT and PC within 30 min of injury, and peak levels of 3-NT at 3 d in the CCI injury model of TBI [18,24]. A close relationship exists between the degree of ROS imbalance and the pathogenesis of TBI [37], and the severity of injury in TBI has been correlated with the degree of ROS-related tissue damage [39]. Via the downstream redox mechanism, the resulting protein oxidative modifications can disrupt protein function, thereby contributing to injury pathology.

The 3-NT is a reaction product of peroxynitrite, a powerful oxidizing and nitrating agent that damages lipids and proteins [40,41]. 3-NT showed immediate accumulation in the injured cortical mitochondria within 30 min, and its biphasic peaks observed at 3 h and 3 d post-injury (Figure 1) suggest that mitochondrial tyrosine residue are highly vulnerable to ROS-RNS, and that PTBI can acutely elevate protein nitration in the mitochondrial membrane. Interestingly, a recent study reported that alterations in the 3-NT profile occurs well before any symptoms appear, and it can be considered a potential target for early diagnosis of neurodegenerative diseases [42]. Neuronal death following intrastriatal injection of 3-nitrotyrosine in rodents has been reported [43]. Early tyrosine nitration can contribute to the susceptibility of neurons to secondary injury post-trauma and neurodegeneration.

Protein carbonylation refers to covalent, nonreversible protein oxidation by the introduction of ketone- and aldehyde groups (i.e., C=O) in a protein sidechain that can react with 2,4-DNPH to form protein-DNP-hydrazones. We observed that proteins with a molecular weight of 38 kDa or more are susceptible to PC adduct formation following PTBI (Figure 2). The significant increase in protein carbonylation observed at 6 h suggests that the biphasic nature of PC adduct formation is later in onset than 3-NT in mitochondrial samples following PTBI, and even continued up to 14 d post-injury. For the most part, oxidatively modified proteins are not repaired and must be removed by proteolytic degradation [44]. Deficiency of proteolysis may cause an increase in the mitochondrial content of oxidatively modified proteins for a longer duration. Observation times longer than 14 d would be necessary to characterize PC elevation and clearance following PTBI.

Increased free radical production due to excess pro-oxidant mechanisms following brain injury can additionally react with nucleic acids, non-membrane proteins, and lipids, altering their structure and function. Moreover, free radicals can also oxidize the core and side chains of proteins, thereby disrupting the function of enzymes, including antioxidants, receptors, neurotransmitters, hormones, and structural proteins via oxidative modification [44,45,46]. Abnormal aggregations of modified mitochondrial proteins may contribute to the bioenergetics crisis with respect to energy homeostasis. Additionally, an increase in tyrosine nitration of SOD and a remarkable decrease in its enzymatic activity has been observed in the brain following trauma [47]. Hence, secondary brain damage following TBI could be the repercussion of an enhanced free radical occurrence coupled with reduced activity of the protective antioxidant defense system, as observed in this study. The oxidative damage to the functions of membrane proteins and the dysregulation of ionic transfers and calcium homeostasis can promote a series of events in the cell, resulting in enhanced ROS production and cellular death, which eventually provokes an apoptotic mechanism leading to neurodegeneration [48]. To evaluate the comprehensive picture, we have been exploring these changes, such as mitochondrial calcium dynamics and neuronal apoptosis, in a separate time course experiments using an identical injury model and time points.

Although an increase in 3-NT and PC expression seen as early as 30 min post-injury is indicative of ROS-induced damage, our earlier study found that the initial phase of mitochondrial bioenergetics declined at 30 min and resolved to baseline levels between 3 h and 6 h in TBI [26]. Thus, most of the mitochondria at 30 min post-injury may still be viable though functionally impaired following the early transient burst in ROS. Ideally, an attempt to pharmacologically protect mitochondria from permanent ROS damage should be initiated within 30 min of injury and then maintained for 7 d to extend the best neuroprotective effect. At 7 d post-injury, both 3-NT and PC expression returned to the Sham levels. In this context, it is worth noting that both 3-NT and PC are nonreversible modifications; the apparent recovery at 7 d may simply be that the damaged mitochondria seen at up to 3 d are no longer part of the sample isolation at 7 d post-injury. The decline in 3-NT and PC levels in response to neuroprotective therapy has been highlighted, which is indicative of the essence of these markers in oxidative stress-related brain and central nervous system pathologies [41,49]. Because blood–brain barrier disruption in PTBI would allow brain-derived protein oxidation products to exit into the plasma, temporal variation in these oxidative products can be explored for TBI biomarkers. This view stems in part from the demonstration that a significant increase in human plasma levels of carbonyl occurs in the first 70 h after severe TBI [50].

During the pathogenesis of TBI, oxidative injury caused by ROS becomes the source of additional oxidative stress, damaging the membranes and protein structures and possibly further promoting ROS propagation, leading to enhanced oxidative impairment [51]. Thus, ROS can induce a vicious cycle that may lead to progressive neuronal death. The most abundant ROS formed in the course of cellular metabolism is the superoxide (O_2_^•−^) free radical, which has been implicated in brain edema and neuronal death [51]. Formation of the O_2_^•−^ is one of the terminal events of several metabolic pathways in the cascade that leads to neuronal death after TBI. It is believed that 1–2% of the oxygen reduced by mitochondria is converted to O_2_^•−^, a free radical chain initiator, at the level of complex I and complex III [52,53]. The first enzymatic reaction in the reduction pathway is the SOD-initiated dismutation of two molecules of O_2_^•−^ when they are converted into hydrogen peroxide (H_2_O_2_), water (H_2_O), and oxygen (O_2_). Thus, SOD acts as a front line of defense against ROS-mediated injury by protecting neurons from a high O_2_^•−^ exposures in TBI. H_2_O_2_, generated following the dismutation of superoxide by SOD, is known to cause degradation/depolymerization of various polymers and must be degraded to prevent oxidative damage. Another first-line of defense antioxidant enzyme, CAT protects cells by detoxifying H_2_O_2_ and plays an imperative role in acquiring tolerance to oxidative stress as an adaptive response [54]. Thus, the extent of oxidative stress is determined in part by the effectiveness of the antioxidant response, involving the enzymes SOD and CAT.

This longitudinal analysis of post-PTBI mitochondria demonstrated a compromised state of SOD2 and CAT (Figure 3 and Figure 4). SODs are a group of metalloenzymes present throughout the body that reduce the superoxide burden in injured tissue. We detected that mitochondria-specific SOD2 expression decreased as early as 30 min post-PTBI, with a maximum depletion observed between 24 h and 3 d, and its level remained diminished until 7 d (Figure 3). Although the time course of SOD2 has not been characterized in a PTBI model, other studies have shown diminishing SOD activity at 24 h that remained low at 7 d post-severe-TBI [33,55]. A decrease in SOD2 levels might be due to its increased utilization to trap the free radicals induced by TBI. Depletion of SOD2 for days after TBI could render injured tissue more vulnerable to increased O_2_^•−^ formation, amplifying post-injury oxidative damage over time. Since the SOD2 expression is downregulated within a few minutes after TBI, replenishing SOD2 activity immediately could be beneficial as an acute treatment of PTBI. The extremely short half-life of SOD2 (~6 min) in circulating blood, and its failure to pass the blood–brain barrier and to be taken up intracellularly, make it challenging to use it for enzyme therapy [56]. However, at the clinical level, a modified enzyme with an increased half-life, such as polyethylene glycol (PEG)-conjugated SOD (PEG-SOD or pegorgotein), has demonstrated promising efficacy in a Phase II clinical trial in TBI patients [57].

More importantly, SOD is one of the most crucial antioxidant enzymes because there is no alternative enzyme to cover its role in detoxifying the elevated levels of O_2_^•−^ generated during oxidative stress following TBI. Therefore, developing strategies to preserve TBI-induced downregulation of SOD activity by supplying SOD mimics that mitigate non-membrane permeable O_2_^•−^ damage would be of prime importance. These mimetics include the orgatein, Mn cyclic polyamines, nitroxides, metalloporphyrins, Mn–salen complexes, and fullerenes, and their chemical properties have previously been well summarized [58].

In contrast to SOD2, the catalase expression increased above Sham levels at 3 h after injury after immediate depletion at 30 min; it peaked at 24 h and remained high at 3 d (Figure 4). At 7 d, CAT levels declined again, approaching 30 min levels, and further depletion was observed at 14 d. CAT, a heme-containing tetrameric protein, is an antioxidant enzyme naturally produced by the body when exposed to oxygen. Although studies have reported an increase in CAT following TBI [33,59], the precise mechanism by which brain injury leads to increased CAT expression is currently unknown. However, studies have demonstrated that nerve growth factor, a neurotrophin released during acute neuroinflammation following mechanical brain injury, can directly increase the expression of CAT in the brain [33,59,60]. Upregulation of CAT for up to 3 d in the sub-acute period may be due to its longer half-life intracellularly. The half-life of catalase has been estimated at approximately 30 h in rodents [61]. An increase in endogenous CAT can be regarded as a compensatory effort made by the mitochondria to self-protect from an oxidative assault. This concept is supported by our observation of CAT expression reversing to below-average levels once other antioxidants’ expression returned to Sham values at 14 d. Under physiological conditions, CAT expression seems to be much lower than other antioxidative enzymes in the brain [62]. Further, the CAT activity in the brain is extremely low compared with other tissues and organs such as the liver and kidney [63]. However, the results of the present study underscore the importance of CAT induction at the transcriptional level as an adaptive mitochondria-protective response during TBI. Without neutralization, membrane-permeable H_2_O_2_ reacts with metal ions and converts into extremely reactive hydroxyl radicals through the Haber–Weiss cycle or Fenton reaction. Hydroxyl radicals have no specific defense antioxidants for its elimination, have a tremendously high oxido-reductive potential, and can easily attack most organic molecules [64]. Since an innate physiological response leads to increased expression of CAT that can quench the dangerous Fenton reaction, supplying precursors of CAT, such as manganese and iron, may boost the intrinsic CAT induction and aid in the recovery from TBI.

Our results documented the upregulation of CAT in mitochondria following severe TBI. However, it remains unclear if the increased expression of the enzyme is through induction of CAT constitutively present in the inner mitochondrial compartments or if it was due to enzyme uptake from peroxisomes or other intracellular organelles. The majority of the intracellular pools of CAT are located in peroxisomes [65], which are in close proximity to mitochondria in the cytosolic environment [66]. These two organelles coordinate their activities and can convey information to each other by releasing biological messengers such as ROS and other metabolites [67]. Nevertheless, gaining more insight into their intricate relationship will provide essential clues in understanding the role of CAT dynamics in TBI pathophysiology. Further studies using the TBI paradigm and describing how mitochondria and peroxisomes coordinate to maintain CAT kinetics during oxidative stress would be of considerable interest.

In addition to SOD2 and CAT antioxidant enzymes, the compartment-specific peroxidases, including peroxiredoxins (PRX) and thioredoxins (TRX), are essential in controlling the cellular thiol pools, and thereby redox-sensitive thiol-containing proteins that are important in various aspects of cellular functions [68]. The thiol-dependent thioredoxin antioxidant system (i.e., Trx-system) is composed of TRX, TRX reductase, and PRX enzymes. In mammalian cells, the cytosolic and mitochondrial Trx-system, in conjunction with the glutathione-glutaredoxin system (i.e., Grx-system), controls the cellular redox environment [69]. Terminal NADPH serves as the cofactor that is used for transferring and reserving reduction potential for the Trx- and Grx-systems. Both of these intracellular antioxidant systems are responsible for H_2_O_2_ and organic peroxides inactivation, yielding water/alcohols and oxygen [70]. TRX has a conserved active site, such as Cys-Pro-Gly-Cys, that acts as an active oxidoreductase and electron donor for some PRX, which are crucial for reducing oxidized peroxides [63]. Likewise, oxidized TRX is reduced by the enzyme TRX reductase using NADPH as a cofactor [71].

The present study revealed that major components of mitochondrial Prx and Trx systems (i.e., PRX-3 and TRX-2 protein expressions) were decreased during the first week of PTBI. The significant depletion of PRX-3 and TRX-2 expression started at 30 min, with maximum depletion observed at 3 d post-injury (Figure 5 and Figure 6). However, TRX-2 remained significantly reduced at 14 d, whereas an elevated level of PRX-3 was observed at this time-point. In line with our observation of significant depletion of H_2_O_2_ neutralizer CAT at 14 d, an increase in PRX-3 at 14 d could be a mitochondrial compensatory effort to continue catalyzing the reduction of H_2_O_2_. The temporal changes in TRX-2 and PRX-3 after brain injury are in concurrence with studies reporting that the Trx-system is depleted in patients with CNS diseases including brain injury [72,73]. Studies have indicated that PRX-3 and TRX-2 offer protection by preserving mitochondrial function and enhancing mitochondrial biogenesis, and may have potential therapeutic value in TBI [74,75,76,77]. Notably, no longitudinal study of either TRX-2 or PRX-3 exists in any of the pre-clinical traumatic brain injury models. This study is the first of its kind to report time-dependent changes in crucial components of the Prx- and Trx-systems post-TBI.

Next, we studied the Grx-system formed by glutathione (i.e., reduced and oxidized forms), glutathione reductase enzyme, and NADPH, which work in close cooperation to protect tissue from the damaging effects of free radicals. Glutathione plays a crucial role in detoxifying peroxides in conjunction with NADPH [78]. In addition to cooperation with glutathione, NADPH plays a decisive role in cellular antioxidation systems by providing reducing equivalents to generate reduced forms of various antioxidant molecules [79]. Antioxidants are oxidized during the detoxification of ROS and are converted into the reduced form by NADPH. This NADPH-induced conversion reactivates the ROS-neutralizing functions of antioxidant molecules. Thus, depletion of redox cofactor NADPH indicates a lack of cofactor supply to replenish/recycle antioxidants following PTBI. Our current results extend our earlier observations [23] by showing that NADPH depletion occurs beyond 24 h of PTBI. We found that NADPH levels significantly decreased as early as 3 h post-injury, and the decreasing trend remained over the course of 7 d post-injury (Figure 7). At 14 d post-PTBI, NADPH content in PTBI was comparable to Sham, which could be indicative of oxidative stress-evoked metabolic adaptation that favors increased NADPH synthesis. Despite its crucial function in redox homeostasis and biosynthetic reactions, the role of NADPH dynamics in TBI pathology still needs to be explored. Interestingly, a significant increase in relative concentrations of NADPH in serum samples of TBI patients has been reported [80], which indirectly supports its biomarker potential following acute TBI. The current study is the first to provide insight into temporal changes in mitochondrial NADPH following severe TBI. From our data, it can be anticipated that exogenous NADPH treatment following PTBI would increase the effectiveness of antioxidant proteins as the scavengers of ROS. Indeed, NADPH supplements have been shown to exert a neuroprotective effect, ameliorating metabolic disturbance and brain injury in an animal model of ischemic stroke [81,82].

In the Grx-system, glutathione is the most abundant and ubiquitously present intracellular thiol antioxidant molecule that plays an essential role against oxidative stress. Approximately 80–85% of the intracellular pool of glutathione is found in the cytosol and around 10–15% of glutathione is present in the mitochondria [83]. In the mitochondria, CAT neutralizes H_2_O_2_, but due to the low CAT content in the brain, glutathione is crucial to maintain the redox balance in CNS. Indeed, neuronal defense against H_2_O_2_ is mediated primarily by glutathione [84]. However, mitochondria are not able to synthesize glutathione and therefore rely on the cytosolic pool for replenishment of their glutathione. Therefore, we investigated glutathione content in an additional two fractions, namely cytosol and tissue homogenate, for better understanding of glutathione dynamics. Because of its ubiquitous presence and principal role in regulating the redox state, a change in glutathione levels is an important marker when assessing oxidative stress responses in TBI.

We found that glutathione level (cytosolic and mitochondrial) depletion occurred as early as 3 h post-injury and remained significantly lower over the course of 3 d. Similarly, glutathione content in homogenate was significantly depleted at the 6 h and 24 h time-points in the PTBI animals compared to Sham (Figure 8). Notably, our results support the possible application of cytosolic glutathione as an indirect measure of oxidative stress when mitochondrial isolation cannot be performed. Consistent with our result, Ansari et al. (2008) observed a decrease in glutathione initially at 3 h post-TBI, and it dropped to the lowest values at 24–48 h post-TBI [38]. At a clinical level, glutathione levels in cerebrospinal fluid (CSF) were found to be significantly decreased from 1 d post-TBI until 7 d post-TBI in infants and children who had suffered a severe TBI when compared to healthy controls [85]. In humans, low glutathione activity immediately post-TBI could contribute to an increase in free radical load and oxidative stress [86], and these circumstances can lead to prolonged neuronal dysfunction [87]. Taken together, our findings indicate that replenishing glutathione would be an excellent therapeutic option to treat or, in some cases, even prevent secondary injury cascades after TBI. However, studies have reported that exogenous glutathione molecules are not effectively transported into most cells and are rapidly degraded in plasma [88]. In contrast, administration of cysteine and glycine, both fundamental amino acids for glutathione biosynthesis that can be taken up by cells, has demonstrated neuroprotective efficacy after mild and severe TBI [1,89,90]. Therefore, the rationale of administering glutathione precursors to replenish the cerebral pool of glutathione in the post-injured brain is well justified.

Collectively, no single marker could be recommended as the gold standard for evaluating redox homeostasis, and elevated oxidative stress responses. An individual marker that only partially reflects the oxidative phenomena would be insufficient. Therefore, we used integrative approaches and selected various redox markers that may give us a more accurate idea and help us to define the oxidant/antioxidant balance in PTBI. We found progressive deterioration of antioxidant capacity initiating within minutes to hours that evolves from a few days to weeks of brain injury. It appears that not all neuronal damage occurs at the same time of primary injury but is markedly exacerbated over time by a complex cascade of pathophysiological events led by oxidative stress during the first few days. Our result also revealed innate adaptive responses of redox elements to protect cells against the dangerous effects of oxidative stress following the brain injury. These compensatory efforts are evidenced by an increased CAT expression during the first few days, and PRX-3 and glutathione expression at 14 d. Thus, adaptation initiated within a week and was partially completed by 2 weeks post-injury.

Our findings offer a few thoughts for designing future TBI therapeutic investigations. Selecting appropriate multiple outcome measures and possibly more time points are critical when designing pre-clinical studies. Otherwise, a single parameter and limited time points may not reliably represent the complex TBI pathology. Explicating the temporal redox hemostasis, we observed that mitochondrial antioxidant capacity was significantly depleted during a period form 24 h through to 3 d. Therefore, our data support the idea of boosting mitochondrial antioxidants as soon as possible from 30 min up to 14 days in order to maintain redox homeostasis following PTBI. We believe that linking oxidative stress pathology with pharmacological approaches will create a clear pathway to validate new TBI therapies. Moreover, the evolving nature of mitochondria-related oxidative stress and various signaling pathways may serve as promising avenues for developing diagnostic biomarkers in clinical and translational medicine within the TBI domain.

Nearly all clinical trials in TBI have failed to show any consistent improvement in patient outcomes. The next decade will witness many clinical trials seeking to translate pre-clinical TBI research discoveries into the clinic. During this bench-to-bed transition, antioxidants should be evaluated with other treatments as therapeutic approaches for TBI. As to the path forward, a rigorous scientific literature search on TBI mechanisms, drug pharmacological properties, and advanced computational algorithms using artificial intelligence technology would be helpful to down-select potential neuroprotective drugs. From this temporal study, we have provided several unique observations which may help to delineate the most beneficial antioxidant therapy in brain injury and what duration of therapy would be most appropriate. Further augmenting the supporting evidence for antioxidants across different species, assessment time points, drug doses, and injury models will further support the evaluation of antioxidants as a therapeutic target for TBI.

In conclusion, according to our knowledge, this study is the first to investigate the comprehensive temporal redox changes attributable to severe penetrating TBI. It was interesting to observe distinct and scorable time-dependent changes in all markers of redox homeostasis. This study demonstrated the dynamic nature of the oxidative stress-led progressive pathological cascade in PTBI developing over hours to days, and it suggests that secondary TBI pathophysiological processes can be valuable targets for neuroprotective interventions. Moreover, redox markers dynamics illustrated a similar trend, and most markers returned back to a normal or exhibited higher levels by 14 d of PTBI. These suggest that the ideal duration for antioxidant treatment strategies following PTBI in rodents may be up to 2 weeks. Therefore, constructing mitochondria-targeted drug delivery systems to regulate mitochondrial functions post-PTBI should be initiated immediately and maintained for 2 weeks or longer to exert the best neuroprotective effects.

### Limitations

One limitation of this study is that the research focused exclusively on rodent penetrating TBI, which means our findings may not be precisely applicable to higher mammal species experiencing mild or moderate TBI. Additionally, the limited volume of mitochondrial yield from the PTBI model posed several constraints on our analysis. The limited number of ROS markers included, along with the absence of lipid peroxidation, may weaken the overall conclusions of our research. Moreover, while we measured the expression of redox markers in mitochondrial samples, we did not conduct serial assessments of these markers in the cytosol or other intracellular compartments, except for glutathione. It is also worth noting that the redox index is ideally represented by the GSH (reduced)/GSSG (oxidized) ratio; however, estimating GSSG requires additional samples, and due to the limited sample volume, this was not accomplished in the current manuscript. The glutathione results in this study reflect total glutathione (GSH) and offer an approximate indication of the redox profile. At this stage, we are unable to compare the antioxidant enzymes activity with their protein expression involved in the TBI pathophysiology due to sample limitations.

## 4. Materials and Methods

### 4.1. Reagents

Mitochondrial isolation and respiration reagents were purchased from Sigma (St. Louis, MO, USA) as described previously [26]. Western blot antibodies were purchased from known vendors [23]. Glutathione and NADPH content were measured using commercial kit methods [23]. A BCA protein assay kit quantifying the protein content in the samples was purchased from Fisher Scientific (Hampton, NH, USA).

### 4.2. Animals and Experimental Design

Adult male Sprague Dawley rats (280–350 g, Charles River Laboratories, Raleigh, VA, USA) were used for this study. Animals were housed individually under a standard 12 h light/dark cycle (lights on at 0600 EST) in a well-ventilated facility and allowed 3 days acclimation to the housing facility before any procedures were performed. Research was conducted under an Institutional Animal Care and Use Committee (IACUC)-approved animal use protocol in an Association for Assessment and Accreditation of Laboratory Animal Care (AAALAC)-accredited facility with a Public Health Services Animal Welfare Assurance and in compliance with the Animal Welfare Act and other federal statutes and regulations relating to laboratory animals.

On the first day of the experiment, age-matched animals were randomized into two groups (i.e., PTBI and Sham) by matching their body weight. Experimenters were blinded to injury group allocations. As illustrated in Table 1 below, experiments were completed using 2 cohorts of animals because of the limited yield of mitochondria. Oxidative stress and antioxidant markers were quantified by Western blot method using Cohort A animals (N = 84 rats). Remaining antioxidants such as glutathione and NADPH levels were quantified by the plate reader method using Cohort B (N = 60 rats). A total of 144 animals were used in the current study.

### 4.3. Penetrating Traumatic Brain Injury Model

All surgical procedures were performed under isoflurane anesthesia (3–5% for induction and 2% for maintenance) and aseptic conditions with careful monitoring of vital physiological signs. The PTBI surgery was performed as described previously [23,91,92]. The PTBI apparatus consists of a specifically designed probe (Kadence Science, Lake Success, NY, USA), a stereotaxic frame (Kopf, Tujunga, CA, USA), and a hydraulic pressure-pulse generator (4B080; MITRE, Bedford, MA, USA). The probe was made of a 20 G stainless steel tube with fixed perforations along one end, which was sealed by a piece of airtight elastic tubing. The probe was secured to the probe holder with the un-perforated end attached to the pulse generator, angled at 50° from the vertical axis. Core body temperature was maintained normothermic (~37 °C) using a heating blanket (Harvard Apparatus, South Natick, MA, USA).

Under isoflurane anesthesia (2%; in air/oxygen mixture), the animal’s head was secured in the stereotaxic device to perform PTBI injury. After a midline scalp incision, a right frontal cranial window (diameter = 4 mm) was created using a dental drill to expose the right frontal lobe (+4.5 mm AP, +2 mm ML to bregma). The PTBI probe was then advanced through the cranial window into the right hemisphere to a depth of 1.2 cm from the brain’s surface. Once the probe was in place, the pulse generator was activated by a computer to release a pressure pulse calibrated to produce a rapid expansion of the water-filled elastic tubing creating an elliptical-shaped balloon (diameter = 0.633 mm) to a volume equal to 10% injury of the total brain volume. This rapid inflation/deflation (duration = 40 milli s) produced a temporary cavity in the brain. After deflation, the probe was immediately retracted from the brain, the cranial opening was sealed with sterile bone wax, and the skin incision was closed with wound clips. Due to the severe nature of the injury, we routinely observed up to 10% mortality in experimental animals after PTBI. All Sham animals underwent craniectomy with no insertion of the PTBI probe.

### 4.4. Mitochondrial Isolation

At the terminal day of each time point, animals were euthanized with CO_2_ (30–70% per minute), rapidly decapitated, and brains were removed and placed in mitochondrial isolation buffer (MIB) at 4 °C. From the ipsilateral hemisphere of PTBI and Sham brains, the frontal cortex and striatum regions were isolated and mixed together for mitochondrial isolation and measurements. This region represents the injury core and perilesional area of the injury where extensive cell death may occur.

All reagents, centrifuges, and tubes were maintained at 4 °C throughout during mitochondrial isolation and completed as previously described in the published method using Ficoll-based mitochondrial isolation procedure [26]. The samples were homogenized in 2 mL of MIB and approximately 60 μL of homogenates were collected in a separate tube for glutathione extraction and estimation. The remaining homogenate samples were centrifuged at 1300× *g* for 3 min to remove cellular debris and nuclei. Then, the pellet was discarded, and the supernatant was further centrifuged at 13,000× *g* for 10 min. After centrifugation, the fraction of supernatants was harvested to obtain the cytosolic fraction for glutathione measurement, as outlined below.

The crude mitochondrial pellet was then subjected to nitrogen decompression to release synaptic mitochondria using a nitrogen cell disruption chamber (model 4639, Parr Instruments, Moline, IL, USA). After the pressure release, samples were placed onto a discontinuous Ficoll gradient and centrifuged at 100,000× *g* for 30 min. The resulting mitochondrial pellets were carefully separated, re-suspended, washed with isolation buffer without EGTA (MIB^−^), and again centrifuged at 10,000× *g* for 10 min. The resultant ultrapure mitochondrial pellets were re-suspended in MIB to achieve a final protein concentration (~10 mg/mL). Absolute protein concentrations of mitochondria (Mito), together with other fractions including homogenate (Homo) and cytosol (Cyto), was determined using a BCA protein assay kit [93,94,95]. After isolation, mitochondria were immediately used or stored at −80 °C for future experiments [23].

### 4.5. Western Blots

At multiple post-injury time points, mitochondrial oxidative stress markers were quantified in mitochondrial samples isolated from PTBI and Sham groups using the Western blot (WB) procedure described previously [25]. On the day of the experiment, samples were diluted to ~1.2–2.4 µg/µL in Milli-Q water, 4× XT Sample Buffer (Bio-Rad Cat # 1610737, Hercules, CA, USA), and XT Reducing agent (Bio-Rad Cat # 1610737). Samples were then heated to 95 °C for 5 min and loaded onto commercially prepared 4–12% Criterion™ XT Bis-Tris Protein Gel (Bio-Rad, Cat # 3450124). Mito samples were loaded at 10 µL per lane to achieve a total of 10–20 µg protein. The Chameleon Duo pre-stained protein ladder (LI-COR, Cat # 928-60000, Lincoln, NE, USA) was included on all gels. Following electrophoresis, samples were transferred to methanol-activated Immobilon FL PVDF membrane (Millipore, Cat # IPFL20200, Bedford, MA, USA) with Trans-Blot^®^ Turbo™ Transfer System (Bio-Rad Cat # 1704150).

After transfer, PVDF membranes were stained for total protein detection using the REVERT™ kit (LI-COR) following the manufacturer’s instructions. Membranes were submerged in Revert stain for 10 min, washed twice (2×) for 1 min each with wash solution (LI-COR, 6.7% (*v*/*v*) glacial acetic acid, 30% (*v*/*v*) methanol, in water), and imaged immediately at 700 nm channel of the Odyssey imaging system (LI-COR). The total protein stain was then removed by 15 min incubation in reversal solution (LI-COR, 0.1 M sodium hydroxide, 30% (*v*/*v*) methanol, in water) followed by 3× quick Tris Buffer Saline (TBS, Fishers, 7447-40-7, Hampton, NH, USA) washes. The membranes were then blocked with Odyssey blocking buffer (LI-COR, 927-50000) and incubated with primary antibodies prepared in the blocking buffer overnight at 4 °C in the orbital shaker.

The primary antibodies used in the experiments were as follows: Catalase (1:1000, ab16731, Abcam, Cambridge, MA, USA); Superoxide Dismutase (1:1000, 06-984, Sigma, St. Louis, MO, USA); Thioredoxin-2 (1:10000, ab185544, Abcam); Peroxiredoxin-3 (1:1000, ab73349, Abcam); 3-NT (1:1000, 06-284, Sigma). The following morning, a corresponding infrared dye secondary antibody (LI-COR IR Dye 800 CW, 1:5000 to 1:25,000) was applied. All incubation and washing steps were performed according to the published method [25]. The intensity of the bands was visualized and quantitated using the Odyssey imaging system (LI-COR).

For ROS-mediated oxidative stress markers such as 3-NT, samples were prepared using 4× Laemmli Sample Buffer (Bio-Rad, Cat # 1610747) and 100 mM dithiothreitol (DTT). For PC detection, samples were prepared in accordance with the manufacturer’s protocol (OxyBlot Kit, Sigma, Cat # S7150), and the PVDF membrane was incubated on the first day with the primary antibody supplied by the OxyBlot kit. All other steps and procedures for 3-NT and PC immunoblotting remain the same.

All blot membranes were analyzed using Image Studio™ Lite (Version 5.0.0). Densitometry of single-band targets and whole lane signals were measured in a manually defined rectangular area, and the background was subtracted using the software’s averaging protocol. The Revert total protein (RTP) stain signal was measured manually by applying a rectangular area at a range of maximum detectable molecular weights in each band [25]. To compare samples on the same gel, relative quantification was achieved by normalizing each target signal to the respective total protein signals’ value of each lane.

### 4.6. Glutathione Estimation

For total glutathione extraction, samples were mixed with 0.5% sulfosalicylic acid (SSA), and a final concentration of 1 µg/1 µL was achieved. Then, acidified subcellular fractions samples (i.e., Homo, Cyto, and Mito) were sonicated in an ice bath for 10 s × 3 times, centrifuged for 10 min at 10,000× *g*, and supernatants collected and stored at −80 °C. Glutathione reduced (GSH) is oxidized with sulfhydryl reagent 5,5′-dithio-bis (2-nitrobenzoic) acid (DTNB) to produce oxidized glutathione disulfide (GSSG) with stoichiometric formation of yellow derivative 5′-thio-2-nitrobenzoic acid (TNB) measurable at 412 nm. GSSG is recycled to GSH by the addition of glutathione reductase in the presence of NADPH. On the day of assay, samples and standards were loaded at 20 µL/well in duplicates and 60 µL of DTNB was added to each well, followed by 60 µL of glutathione reductase. Finally, 60 µL of NADPH was added to generate the TNB product. The rate of TNB formation is quantified at 412 nm for 10 min and is proportional to the total glutathione present [96]. All values are represented as percent change (% change vs. Sham).

### 4.7. NADPH Assay

The amount of NADPH in mitochondrial samples was measured by using a commercial quantitation kit (Sigma, Cat # MAK312). NADPH extraction was carried out using 10 µL of mitochondrial samples in NADPH extraction buffer to achieve a final sample concentration of 1 µg/1 µL. The sample mixtures were centrifuged for 10 min at 10,000× *g*, and the supernatant was collected and stored at −80 °C. On the day of the NADPH assay, 60 µL aliquot of samples were incubated in the dark at 60 °C for 30 min. Heating decomposes the oxidized form of NADP (i.e., NADP^+^) while not affecting the reduced form (i.e., NADPH). During the incubation, standards were prepared per the manufacturer’s protocol and loaded in 96-well plates in duplicates. After heating, the samples were kept on ice, briefly centrifuged, and loaded in duplicates at 25 µg/well. We chose the sample dilution after testing several dilutions to ensure the readings were within the linear range of the NADPH standard curve. First, NADPH cycling buffer was added to each well, followed by a NADPH cycling enzyme mix and a high-sensitivity probe. NADPH concentration was determined by measuring the fluorescence (Ex λ = 535 nm/Em λ = 587 nm) in a microplate reader after an hour of incubation [97]. The results were expressed as percent change (% change vs. Sham).

### 4.8. Statistical Analysis

Results are expressed as mean ± standard error of the mean (SEM). Considering the blot-to-blot variations in the expression of protein signals, including the Sham, to minimize errors, differences between the Sham and PTBI groups were evaluated by a two-tailed *t*-test, assuming equal variances at each time point. To compare statistical significance, we randomized N = 6 animals/group (PTBI and Sham) for oxidative stress and antioxidant measurements using WB experiments. Additionally, for glutathione and NADPH content estimation by plate reader assays, we used N = 3–6 animals/group (PTBI and Sham). Statistical comparisons were conducted using Prism-GraphPad software (Version 8), and statistical significance was defined as *p*-value * *p* < 0.05.

## Figures and Tables

**Table 1:** Total Number of animals used.

Outcome Measure	N = Number of Animals Used at Each Time Point × 2 Groups (PTBI and Sham)									
	Methods	Cohorts	30 min	3 h	6 h	24 h	3 d	7 d	14 d	Total
Oxidative Stress & Antioxidants	Western Blot	A	N = 6	N = 6	N = 6	N = 6	N = 6	N = 6	N = 6	84
Glutathione & NADPH	Plate Reader	B	N = 3–6	N = 3	N = 3–6	N = 3–6	N = 3	N = 3	N = 3	60
										N = 144

## Data Availability

The data presented in this study are available on request from the corresponding author. The raw data currently not openly accessible due to WRAIR clearance requirement.

## Abbreviations

TBI: Traumatic brain injury, PTBI: Penetrating Traumatic Brain Injury, ATP: Adenosine 5′ Triphosphate, ROS: Reactive Oxygen Species, O_2_^•−^: Superoxide radical, ^•^OH: hydroxyl radical, H_2_O_2_: Hydrogen Peroxide, PC: Protein Carbonyl, 3-NT: 3-Nitrotyrosine, NADPH: Nicotinamide Adenine Dinucleotide Phosphate Reduced, SOD: Superoxide Dismutase, SOD2: Superoxide Dismutase 2, CAT: Catalase, PRX-3: Peroxiredoxin-3, TRX-2: Thioredoxin-2, CNS: Central Nervous System, WB: Western blot.
